# 

**Published:** 1894-03-31

**Authors:** 


PRICE TWOPENCE.

No. 392, Vol. XV.—March 31st, 1894.

[Registered at the General Post Office as a Newspaper for transmission in the United Kingdom and Abroad.]

WITH EXTRA NURSING SUPPLEMENT.

Per Annum, 8s. 6d., post free.

Monthly Parts, 6d.; post free, 8d.

Ditto per Annum, 8s., post free.

LIBRARY
Indexed
S. G. O.

### THE HOSPITAL

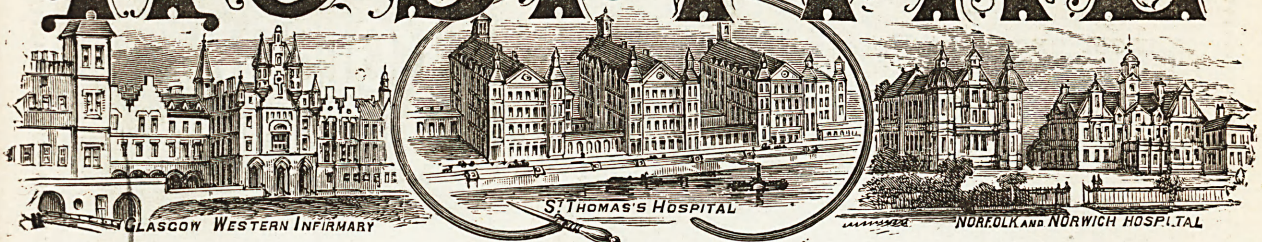

No. 392. Vol. XV. WITH EXTRA NURSING SUPPLEMENT. SATURDAY, MARCH 31st, 1894.

